# Black seed assisted synthesis, characterization, free radical scavenging, antimicrobial and anti-inflammatory activity of iron oxide nanoparticles

**DOI:** 10.1186/s12906-024-04552-9

**Published:** 2024-06-20

**Authors:** Rajeshkumar Shanmugam, M. Tharani, Shahabe Saquib Abullais, Santosh R. Patil, Mohmed Isaqali Karobari

**Affiliations:** 1 Nanobiomedicine Lab, Centre for Global Health Research, Saveetha Medical College and Hospitals, Saveetha Institute of Medical and Technical Sciences, Chennai, Tamil Nadu 602105 India; 2 Department of Periodontics, College of Dentistry, King Khalid University, Abha, 62529 Saudi Arabia; 3 Department of Oral Medicine and Radiology, Chhattisgarh Dental College & Research Institute, Chhattisgarh, India; 4 Department of Dental Research, Center for Global Health Research, Saveetha Medical College and Hospitals, Saveetha Institute of Medical and Technical Sciences, Chennai, Tamil Nadu 602105 India; 5 Department of Restorative Dentistry & Endodontics, Faculty of Dentistry, University of Puthisastra, Phnom Penh, 12211 Cambodia

**Keywords:** Iron oxide nanoparticles, Anti-inflammatory, Characterization, Antimicrobial, Antioxidant

## Abstract

Iron nanoparticles comprise a significant class of inorganic nanoparticles, which discover applications in various zones by prudence of their few exciting properties. This study achieved the green synthesis of iron oxide nanoparticles (IONPs) by black cumin seed (*Nigella sativa*) extract, which acts as a reducing and capping agent. The iron nanoparticles and black cumin extract were synthesized in three different concentrations: (01:01, 02:04,01:04). UV-visible spectroscopy, XRD, FTIR, and AFM characterized the synthesized iron oxide nanoparticles. UV-visible spectra show the maximum absorbance peak of 01:01 concentration at 380 nm. The other concentrations, such as 02:04, peaked at 400 nm and 01:04 at 680 nm, confirming the formation of iron oxide nanoparticles. AFM analysis reveals the spherical shape of iron oxide nanoparticles. The XRD spectra reveal the (fcc) cubic crystal structure of the iron oxide nanoparticles. The FTIR analysis’s peaks at 457.13, 455.20, and 457.13 cm-1 depict the characteristic iron nanoparticle synthesis. The black cumin extract-mediated iron oxide nanoparticles show substantial antibacterial, antifungal, antioxidant and anti-inflammatory activity in a dose-dependent manner.

## Introduction

Antimicrobial resistance (AMR) is a significant global health concern, with bacteria evolving to resist the effects of antibiotics, making it difficult or impossible to treat infections caused by these bacteria with standard antibiotic therapies [1]. The overuse and misuse of antibiotics are the main contributing factors to AMR, while environmental stress, unhygienic conditions, illiteracy, and unawareness also contribute. The slow and costly development of new antibiotics has lagged behind the emergence of antibiotic-resistant bacteria, and the overuse of antibiotics leads to negative consequences [2].

Nanotechnology offers a promising solution to combat AMR. Nanoparticles can be engineered to disrupt bacterial cell walls or membranes, effectively eliminating resistant strains [3]. Additionally, nanoscale devices enable the real-time monitoring of bacterial populations, allowing for the early detection of resistance emergence. Nanotechnology, along with evolutionary theory, offers a powerful approach to combat AMR, offering new avenues for the development of effective treatments and the preservation of our antibiotic arsenal [4].

Iron oxide nanoparticles possess unique properties that make them effective in combating antimicrobial resistance. These properties include being biocompatible, biodegradable, and having unique non-toxic magnetic properties [5]. Additionally, iron oxide nanoparticles can interact with bacterial cell membranes via electrostatic contact, causing harmful oxidative stress in the bacterium, leading to their antibacterial activity. The nanoparticles have shown significant improvement in antibacterial activity against bacterial isolates, especially when combined with other compounds like polyethylene glycol and gentamicin, resulting in an increased diameter of the inhibition zone against various bacteria strains [6]. Iron oxide nanoparticles have also been found to be effective against biofilms produced by isolated bacteria, further enhancing their antimicrobial properties.

Moreover, the multifunctional magnetic iron oxide nanoparticles offer diverse synthetic approaches and surface modifications, making them cytotoxic towards various biomedical and industrial applications. These nanoparticles can disrupt bacterial cell walls, cause leakage of cytoplasmic components, and generate reactive oxygen species that disrupt bacterial functions, ultimately leading to cell death [7].The shape of iron oxide nanoparticles, whether rod-shaped or sphere-shaped, can also impact their toxicity to murine macrophage cells, with rod-shaped nanoparticles being more toxic [8].

Green synthesis of nanoparticles is an eco-friendly and sustainable approach to combat antimicrobial resistance. This method utilizes plant extracts, bacteria, and fungi to produce nanoparticles, which eliminates the need for toxic chemicals used in traditional synthesis methods [9]. Among these, plant extracts are particularly desirable as they offer several advantages, including cost-effectiveness, scalability, and the ability to produce nanoparticles with unique physicochemical properties [10, 11].

Black cumin seed extract, also known as *Nigella sativa*, has shown promising antibacterial activity against various clinical isolates of bacteria resistant to a number of antibiotics. The oil of black cumin has demonstrated pronounced dose-dependent antibacterial activity, which is more effective against Gram-positive bacteria than Gram-negative bacteria. Among Gram-positive bacteria tested, Staphylococcus aureus, S. epidermidis, other coagulase-negative Staphylococci, and Streptococcus pyogenes were sensitive to the oil, while Enterococcus faecalis and Streptococcus agalactiae were resistant [12]. Among Gram-negative bacteria tested, only Pseudomonas aeruginosa was sensitive to the oil, while Acinetobacter baumannii, Citrobacter freundii, Klebsiella pneumoniae, Proteus mirabilis, P. vulgaris, and Vibrio cholerae were insensitive [4].The antibacterial activity of black cumin seed extract is attributed to its active ingredients, thymoquinone and melanin, which disrupt bacterial membranes and cause leakage of cytoplasmic components, ultimately leading to cell death [13]. The extract has also shown antibacterial synergism with streptomycin and gentamicin and additive antibacterial action with spectinomycin, erythromycin, and other antibiotics [14].Black cumin seed extract has also been reported to have antifungal, antiviral, and immune-stimulating properties, making it a potential candidate for the development of new antimicrobial agents [15].

In this study, black cumin seed extract was utilized as a dual-functioning agent for both reducing and stabilizing the synthesis of iron oxide nanoparticles. Following synthesis, the nanoparticles underwent comprehensive characterization using various analytical techniques. UV-Visible spectroscopy, Fourier Transform Infra-red spectroscopy (FT-IR), X-Ray Diffraction analysis (XRD), Atomic Force Microscopy (AFM). Beyond characterization, the bioactivity of the synthesized nanoparticles was investigated through in vitro assays. Their antioxidant potential was assessed, along with evaluations of antimicrobial and anti-inflammatory activities.

## Materials and methods

### Preparation of seed extract

Black cumin seeds (Nigella sativa) were procured from a local supermarket in the vicinity of Poonamallee. The seeds were ground into a fine powder using a laboratory-grade grinder. Subsequently, 1 gram of the powdered black cumin seeds was accurately weighed and dissolved in 100 mL of distilled water to prepare an aqueous extract. The resulting mixture was subjected to heating using a controlled heating mantle set at 70 °C for a duration of 15 min. Following heating, the mixture was carefully filtered through Whatman No. 1 filter paper to remove any insoluble particles, yielding a clear filtrate. The 80 mL filtrate, constituting the aqueous extract of black cumin seeds, was promptly cooled and stored under refrigeration conditions to preserve its integrity for further experimental investigations [11].

### Synthesis and characterization of nanoparticles

Ferric chloride hexahydrate (FeCl3·6H2O) served as the precursor for the synthesis of iron oxide nanoparticles (IONPs). Black cumin seed extract was introduced dropwise into solutions of ferric chloride hexahydrate at varying molar ratios of 0.25:1, 0.5:1, and 1:1, denoted as 01:04, 02:04, and 01:01, respectively. The reaction mixture was subjected to continuous stirring using a magnetic stirrer set at 800 rpm for a duration of 48 h, facilitating the formation of IONPs.Following the synthesis, the resultant iron nanoparticles were separated from the reaction mixture via centrifugation at 8000 rpm for 10 min, yielding pellets containing the desired nanoparticles. Subsequently, the obtained pellets underwent purification through sequential washing steps using ethanol and distilled water, repeated 2–3 times to ensure removal of any residual impurities or unreacted components.

The purified pellet, comprising the synthesized iron oxide nanoparticles, was then stored under refrigeration conditions to maintain its stability and integrity for subsequent utilization in further experimental investigations.

### Characterization of Iron oxide nanoparticles

The green-synthesized iron oxide nanoparticles at various concentrations were subjected to Fourier-transform infrared (FT-IR) analysis to elucidate the functional groups present in the biosynthesized nanoparticles. X-ray diffraction (XRD) analysis was employed to confirm the crystalline nature of the nanoparticles. Additionally, atomic force microscopy (AFM) was utilized to investigate the three-dimensional structure of the biosynthesized iron oxide nanoparticles. Furthermore, the reducing and stabilizing capabilities of black cumin seed extract in the synthesis of iron oxide nanoparticles from ferric chloride hexahydrate were assessed using UV-visible spectrophotometry.

### Biomedical applications of Iron oxide nanoparticles

#### Antibacterial activity

The antibacterial activity was assessed using the agar well diffusion technique. Cultures of Escherichia coli and Streptococcus species were freshly inoculated in sterile Hi-Veg broth medium and incubated for 18 h in an orbital shaker at 120–150 rpm to obtain log-phase growth. Mueller Hinton agar plates were prepared as per standard protocol.

The efficacy of black cumin seed extract-mediated iron oxide nanoparticles at varying concentrations against the bacterial pathogens was evaluated. Both Escherichia coli (Gram-negative) and Streptococcus species (Gram-positive) were evenly spread on the surface of each sterile Mueller Hinton agar plate. Four wells were then created using a gel puncher in each plate. These wells were loaded with different volumes (25, 50, 100, and 150 µL) of biosynthesized iron nanoparticles, while the fourth well contained black cumin seed extract. Additionally, a standard antibiotic (Tetracycline) was placed at the center of each petri-plate to serve as a positive control. The plates were subsequently incubated at 37ºC for 24 h to allow for bacterial growth and the diffusion of antibacterial agents. After the incubation period, the plates were visually inspected, and the diameter of the zones of inhibition around each well was measured to assess the antibacterial activity of the synthesized iron nanoparticles.

### Antifungal activity

The antifungal activity was assessed using the agar well diffusion technique. Fresh fungal cultures of Aspergillus niger and Aspergillus flavus were inoculated into sterile Hi-Veg broth medium and incubated for 18 h in an orbital shaker at 120–150 rpm to achieve optimal growth. Sabouraud’s dextrose agar (SDA) plates were prepared according to standard procedures.

The efficacy of black cumin seed extract-mediated iron oxide nanoparticles at various concentrations against fungal pathogens was investigated. Each fungal pathogen was evenly spread on the surface of sterile SDA plates. Using a gel puncher, four wells were created in each plate. The first three wells were loaded with different volumes (25 µL, 50 µL, and 100 µL) of biosynthesized iron nanoparticles, while the fourth well contained black cumin seed extract. Additionally, a standard antifungal agent (Fluconazole) was placed at the center of each Petri plate to serve as a positive control.The plates were then incubated at 28 °C for 48 h to allow for fungal growth and diffusion of antifungal agents. After the incubation period, the plates were visually examined, and the diameter of the zones of inhibition around each well was measured to assess the antifungal activity of the synthesized iron nanoparticles.

### Antioxidant activity

#### Hydrogen peroxide scavenging activity

The scavenging activity of black cumin seed extract towards hydrogen peroxide radicals was evaluated. A 40 mM hydrogen peroxide (H2O2) solution was prepared in phosphate buffer at pH 7.4, and its concentration was determined by measuring the absorbance at 560 nm using a UV-double beam spectrophotometer. Subsequently, 0.1 mg/mL of black cumin extract was added to the hydrogen peroxide solution, and the absorbance was measured again at 560 nm using a UV spectrophotometer against a blank solution containing phosphate buffer without hydrogen peroxide.

$$\% \,{\text{scavenging activity  =  }}{{{\text{1 - Abs }}\left( {{\text{standard}}} \right)} \mathord{\left/ {\vphantom {{{\text{1 - Abs }}\left( {{\text{standard}}} \right)} {{\text{Abs }}\left( {{\text{control}}} \right){\text{x 100}}}}} \right. \kern-\nulldelimiterspace} {{\text{Abs }}\left( {{\text{control}}} \right){\text{x 100}}}}$$

Abs control was the absorbance of the control (without extract) at 560 nm; Abs sample was the absorbance in the presence of the extract at 560 nm. The experiment was repeated in triplicate.

### Nitric oxide scavenging activity

Sodium nitroprusside (SNP) was utilized as a nitric oxide (NO) radical generator in this study. Upon reaction with oxygen in an aqueous solution, sodium nitroprusside yields nitrite ions. A 100 µL aliquot of sodium nitroprusside solution (5 mM, pH 7.4) was added to 100 µL of various concentrations (10, 20, 30, 40, and 50 µL) of black cumin extract-mediated iron oxide nanoparticles and ascorbic acid (50–1000 µg/mL). The reaction mixture was incubated at 25 °C for 30 min. A control solution devoid of test nanoparticles was also prepared.After incubation, 1.5 mL of the reaction solution was diluted with 1.5 mL of Griess reagent (composed of 1% sulfanilamide, 2% phosphoric acid, and 0.1% N-1-naphthyl ethylene diamine dihydrochloride). Subsequently, the absorbance of the mixture was measured at 546 nm using a Shimadzu UV-2450 spectrophotometer, following the method outlined by Rajeshwari et al. (2015).

$$\% \;{\text{inhibition  =  }}\frac{{{\text{Absorbance of contral  -  Absorbance of sample}}}}{{{\text{Absorbance of contral}}}} \times 100$$

### DPPH assay

The antioxidant activity of biogenically synthesized iron oxide nanoparticles was evaluated using the DPPH assay. Various concentrations (2–10 µg/mL) of black cumin seed extract-mediated iron oxide nanoparticles were mixed with 1 mL of 0.1 mM DPPH solution in methanol and 450 µL of 50 mM Tris-HCl buffer (pH 7.4), followed by incubation for 30 min. Subsequently, the reduction in the quantity of DPPH free radicals was assessed based on the absorbance measured at 517 nm using a spectrophotometer. Butylated hydroxytoluene (BHT) was used as a control. The percentage of inhibition was calculated using the following equation:

$$\% \;{\text{inhibition  =  }}\frac{{{\text{Absorbance of contral  -  Absorbance of test sample}}}}{{{\text{Absorbance of contral}}}} \times 100$$

### Anti-inflammatory activity

#### Albumin denaturation assay

The anti-inflammatory activity of black cumin-mediated iron oxide nanoparticles was assessed using a method adapted from Muzushima and Kabayashi, with specific modifications as proposed by Pratik Das et al. (2019). Iron oxide nanoparticles of varying concentrations (10, 20, 30, 40, and 50 µL) were added to 0.45 mL of bovine serum albumin (1% aqueous solution). The pH of the mixture was adjusted to 6.3 using a small volume of 1 N hydrochloric acid. These samples were then incubated at room temperature for 20 min followed by heating at 55 °C in a water bath for 30 min. Subsequently, the samples were cooled, and the absorbance was measured spectrophotometrically at 660 nm. Diclofenac sodium was used as the standard reference. Dimethyl sulfoxide (DMSO) served as the control. The percentage of protein denaturation was determined using the following equation:

$$\% \;{\text{inhibition  =  }}\frac{{{\text{Absorbance of contral  -  Absorbance of sample}}}}{{{\text{Absorbance of contral}}}} \times 100$$

### Egg-albumin denaturation assay

The protein denaturation assay utilized fresh hen’s egg albumin as the substrate. The reaction mixture consisted of 0.2 mL of fresh egg albumin, 2.8 mL of phosphate-buffered saline at pH 6.4, and 2 mL of different concentrations (10 µL, 20 µL, 30 µL, 40 µL, 50 µL) of black cumin-mediated iron nanoparticles. Double distilled water was included as a control. The reaction mixture was incubated at 37 ± 2 °C in an incubator for 15 min, followed by heating at 70 °C for 5 min. After cooling to room temperature, the absorbance was measured at 660 nm using a spectrophotometer. Diclofenac sodium was used as the standard control reference (Alamgeer et al., 2015).The percentage of protein denaturation was determined utilizing the following equation,

$$\% \;{\text{inhibition  =  }}\frac{{{\text{Absorbance of contral  -  Absorbance of sample}}}}{{{\text{Absorbance of contral}}}} \times 100$$

## Results and discussion

**Fig. 1:**
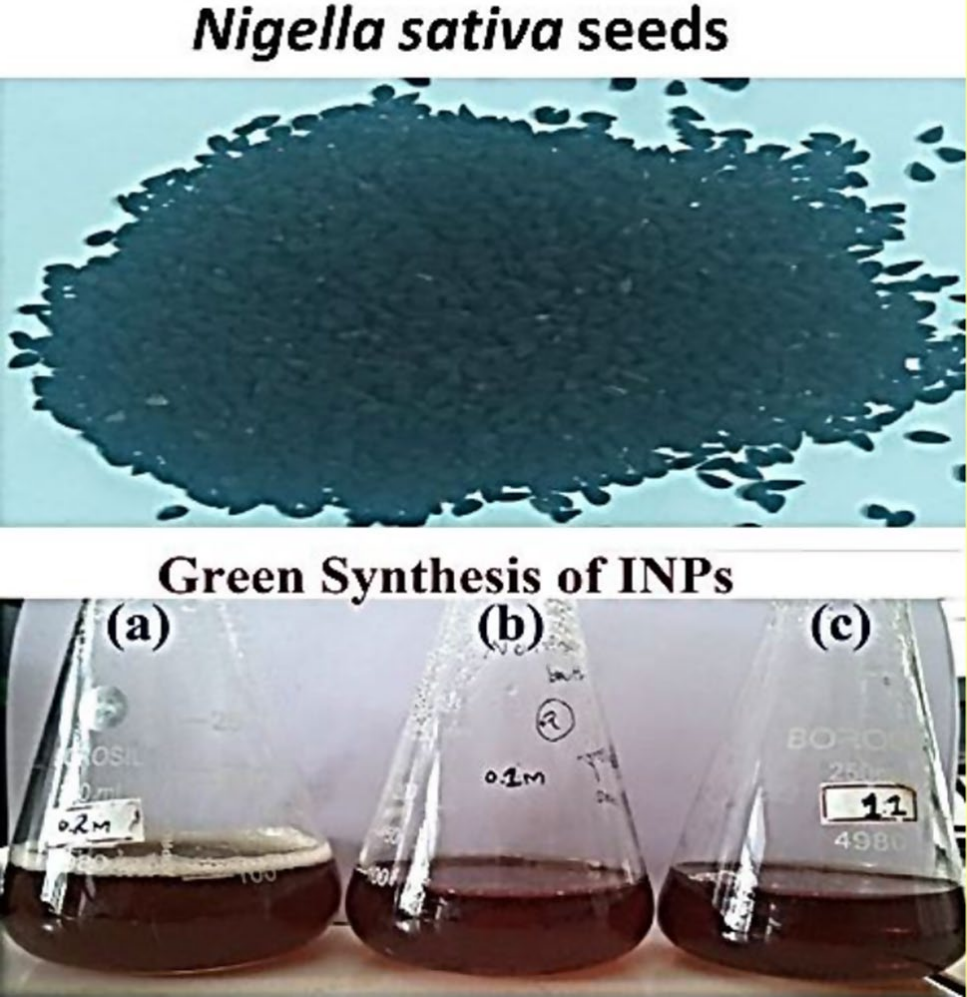
Visual observation of black cumin extract mediated iron nanoparticles (**a**) 001:01 (**b**)01:04 (**c**) 02:04

The green synthesis of iron oxide nanoparticles was initially confirmed through visual observation, as described by Rajeshkumar et al.2017 [16]. The reaction mixture, containing the precursor solution along with the reducing and stabilizing agent, exhibited a distinct color change. Specifically, the biogenic iron oxide nanoparticles, synthesized at three different concentrations in separate flasks, displayed a transition from the initial brown color to a darker shade of brown. This observable change in color is indicative of the reducing and capping abilities of the black cumin seed extract, as depicted in Fig. 1.

The green synthesis of iron oxide nanoparticles using seed extracts has several advantages over traditional synthesis methods, including being eco-friendly, cost-effective, and producing nanoparticles with unique physicochemical properties. These nanoparticles have shown promising results in combating antimicrobial resistance by disrupting bacterial cell membranes, causing leakage of cytoplasmic components, and generating reactive oxygen species that disrupt bacterial functions, ultimately leading to cell death [17, 18].

### UV-Visible spectroscopy

**Fig. 2:**
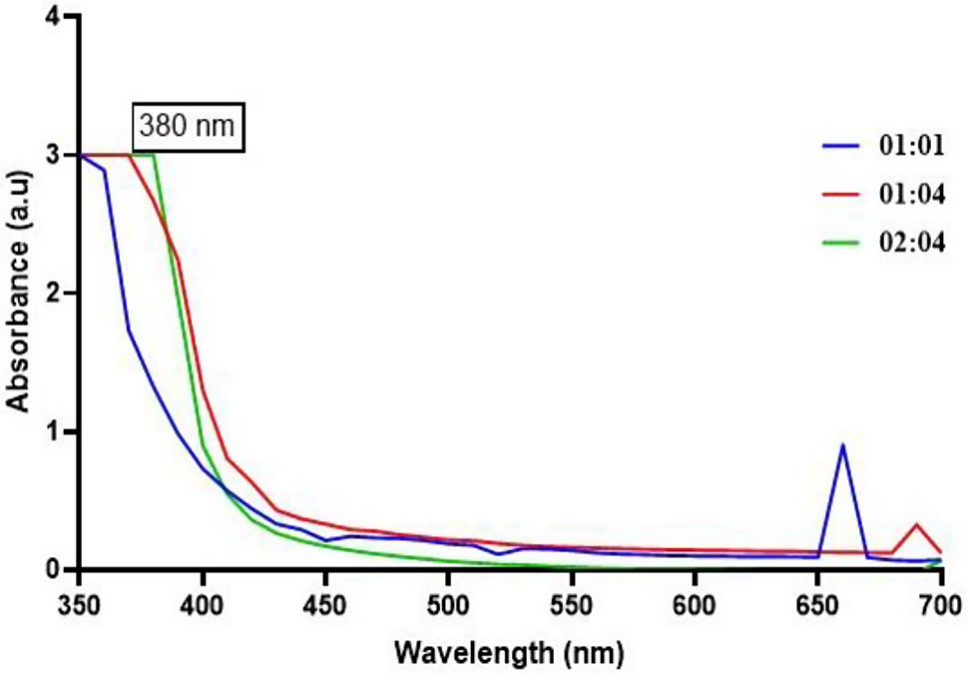
UV-vis spectroscopic analysis of Iron NPs synthesized using *Nigella sativa*

The green synthesis of iron oxide nanoparticles using seed extracts has been reported to produce nanoparticles with unique physicochemical properties, including surface plasmon resonance (SPR) vibration peaks. UV-visible spectroscopy has been used to analyze the synthesis of iron oxide nanoparticles at specific intervals, providing details about the SPR vibration peaks [19]. The UV spectra depicted in Fig. 2 show the wavelength range between 350 and 650 nm, with the iron nanoparticles synthesized using three different concentrations of the precursor ferric chloride solution. The black cumin seed extract-mediated iron oxide nanoparticles of 01:01 concentration showed an absorption peak at 380 nm, while the other concentrations, such as 02:04, peaked at 400 nm and 01:04 at 680 nm. The peak obtained by 01:04 M concentration indicates the phenolic acid constituents of the black cumin seed extract, while the UV absorption peak at 380 nm of 01:01 concentration correlates with the previous research work encompassing the synthesis of iron oxide nanoparticles using proanthocyanidin [17]. The SPR vibration peaks of iron oxide nanoparticles are influenced by their size, shape, and surface functionalization, and these properties can be tailored to achieve specific antimicrobial effects [20, 21].

The absorption peak at 380 nm in the UV spectra of the 01:01 concentration of iron oxide nanoparticles is significant because it indicates the presence of iron oxide nanoparticles. The UV spectra of iron oxide nanoparticles typically show intense absorption between ~ 320 and ~ 420 nm, which corresponds to the band gap energy of about 2.1 eV. The absorption peak at 380 nm in the UV spectra of the 01:01 concentration of iron oxide nanoparticles is consistent with the reported spectra of iron oxide nanoparticles [22]. This peak is attributed to the surface plasmon resonance (SPR) vibration of the iron oxide nanoparticles, which is influenced by their size, shape, and surface functionalization [23]. The SPR vibration peaks of iron oxide nanoparticles are an important parameter for understanding their physicochemical properties and their potential applications in various fields, including biomedicine and catalysis [24].

### FT-IR

**Fig. 3:**
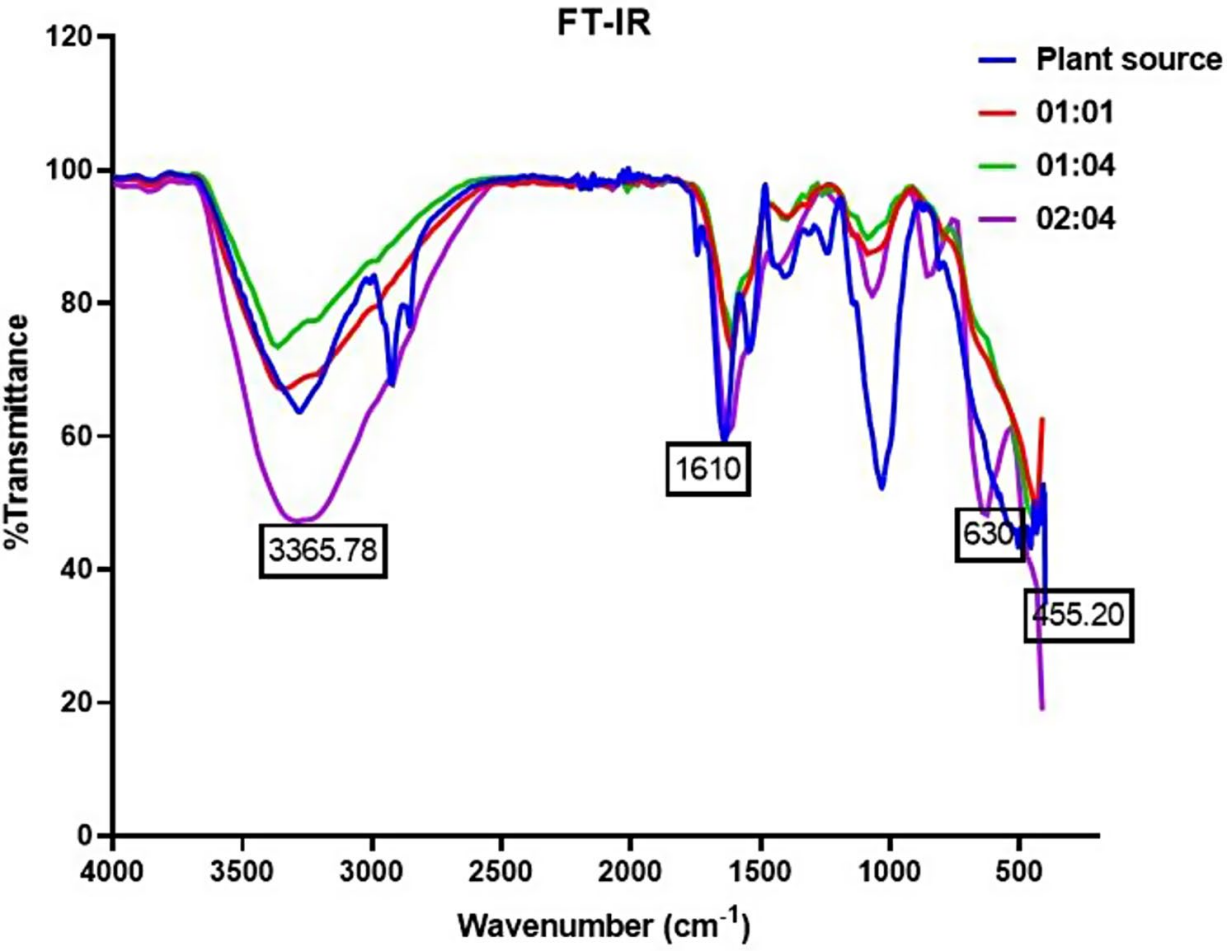
FT-IR spectrum of INPs at different concentrations

The FT-IR analysis of black cumin seed extract-mediated iron oxide nanoparticles revealed the presence of various functional groups, including secondary amines, alkanes, ketones, alkenes, nitro groups, and ethers. The green synthesized iron nanoparticles exhibited major absorbance peaks at 3362.28, 3365.78, and 3259.70 cm-1, indicating the presence of carboxylic acids. The peaks at 457.13, 455.20, and 457.13 cm-1 confirmed the presence of magnetite iron nanoparticles (Fig. 3). The FT-IR results of Ficus carica fruit extract-mediated iron nanoparticles seem concordant with this research work, indicating the potential of plant extracts in the green synthesis of iron oxide nanoparticles [25]. The FT-IR analysis is a valuable tool for identifying the functional groups in nanoparticles, which can provide insights into their surface chemistry and potential applications [24].

The presence of magnetite iron nanoparticles in the FT-IR spectra of iron oxide nanoparticles is significant as it confirms the successful synthesis of these nanoparticles. In FT-IR spectra, the characteristic absorption bands associated with magnetite iron nanoparticles provide evidence of their presence and chemical composition [26]. The specific absorption bands observed in the FT-IR spectra, such as the wide strong absorption band between 580 and 630 cm − 1 and the Fe–O bond peak at 576 cm − 1, are indicative of the presence of magnetite iron nanoparticles. Additionally, the FT-IR spectra reveal other functional groups and interactions, such as C–H stretching vibrations, N–H stretching vibrations, amid groups, Si–O stretching vibrations, and C–N stretching vibrations, which further characterize the iron oxide nanoparticles and their surface modifications [27]. Overall, the FT-IR analysis provides valuable information about the chemical composition, surface functionalization, and structural properties of the synthesized iron oxide nanoparticles, confirming the successful formation of magnetite iron nanoparticles in the sample [28].

### XRD

**Fig. 4:**
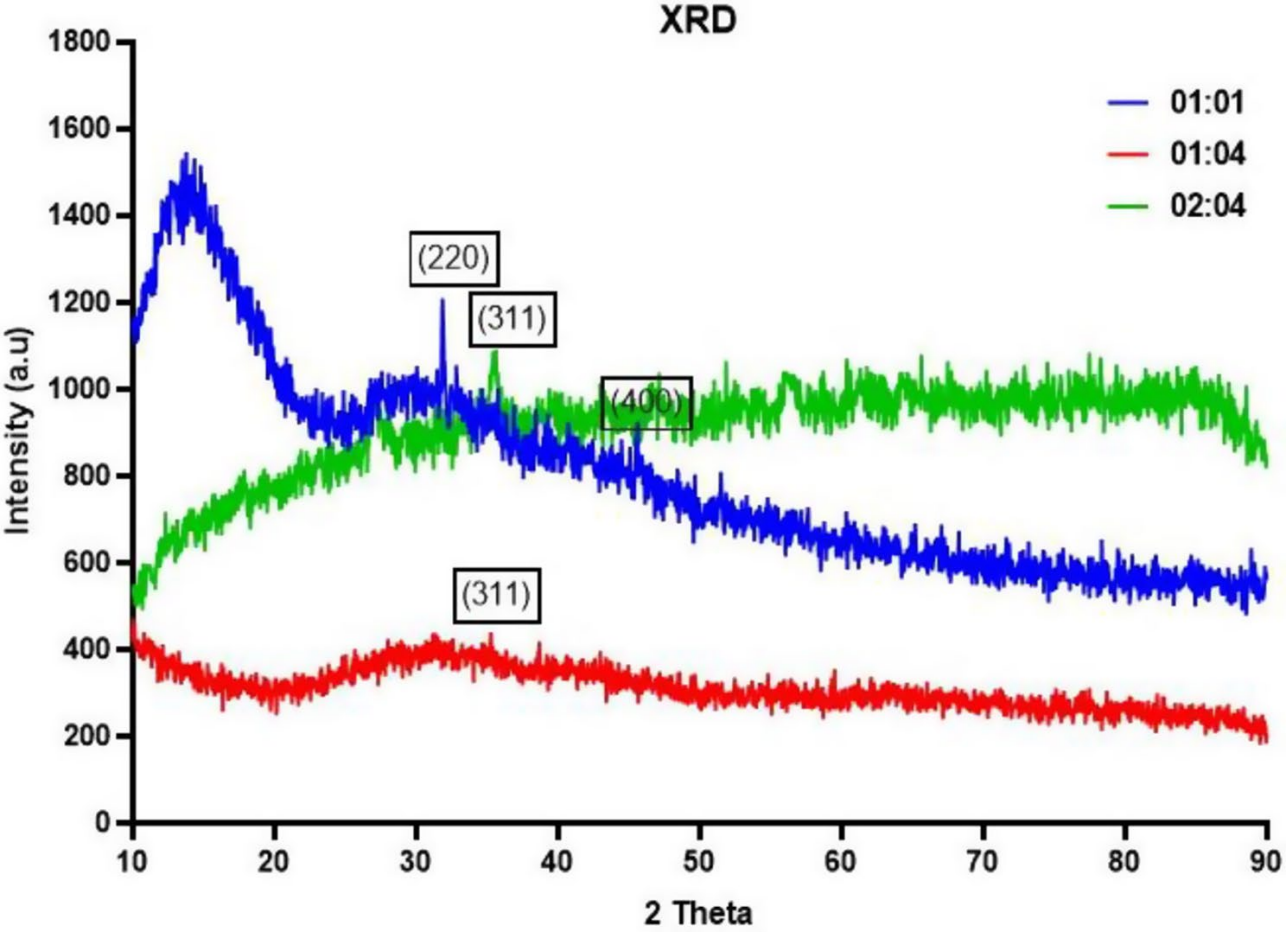
XRD pattern of Iron oxide nanoparticles synthesized using *Nigella sativa*

The biosynthesized iron oxide nanoparticles underwent XRD analysis, with their spectra presented in Fig. 4. X-ray diffraction patterns for black cumin-mediated iron nanoparticles were recorded within a range of 10º to 80º at two theta angles. The XRD analysis detected peaks at specific 2θ angles, namely 31.8º, 35.2º, and 35.4º.These peaks were assigned to crystallographic planes (220), (311), and (400) of the iron oxide nanoparticles which was referenced against JCPDS card No: (89–586).

Utilizing the Debye-Scherrer equation, the size of the iron nanoparticles was determined to be 16 nm for the 01:01 concentration [29], 12 nm for the 01:04 concentration [30]and 4 nm for the 02:04 concentration [31].Despite variations in size, all three concentrations exhibited similar crystalline characteristics.

The crystalline characteristics of iron oxide nanoparticles can significantly affect their properties, particularly their magnetic, structural, and size-dependent properties. The crystal structure of iron oxide nanoparticles can influence their magnetic behavior, such as superparamagnetism, magnetization values, and magnetic interactions between nanoparticles. For instance, the different polymorphs of iron oxide nanoparticles, such as magnetite (Fe3O4), maghemite (γ-Fe2O3), and hematite (α-Fe2O3), have distinct magnetic properties due to their different crystal structures [32] .

The size of iron oxide nanoparticles can also affect their magnetic properties, as smaller nanoparticles exhibit superparamagnetism, while larger nanoparticles may have more complex magnetic behavior. The size of iron oxide nanoparticles can be controlled during synthesis, and the choice of synthesis method can influence the size distribution and morphology of the nanoparticles [33].

The crystalline structure of iron oxide nanoparticles can also affect their optical and structural properties. For example, the crystal structure and size of iron oxide nanoparticles can influence their absorption and scattering of light, which can be important for their use in applications such as biomedical imaging and biosensing. Additionally, the crystalline structure and defects in iron oxide nanoparticles can affect their mechanical properties, such as hardness and elasticity [34].

### AFM

**Fig. 5:**
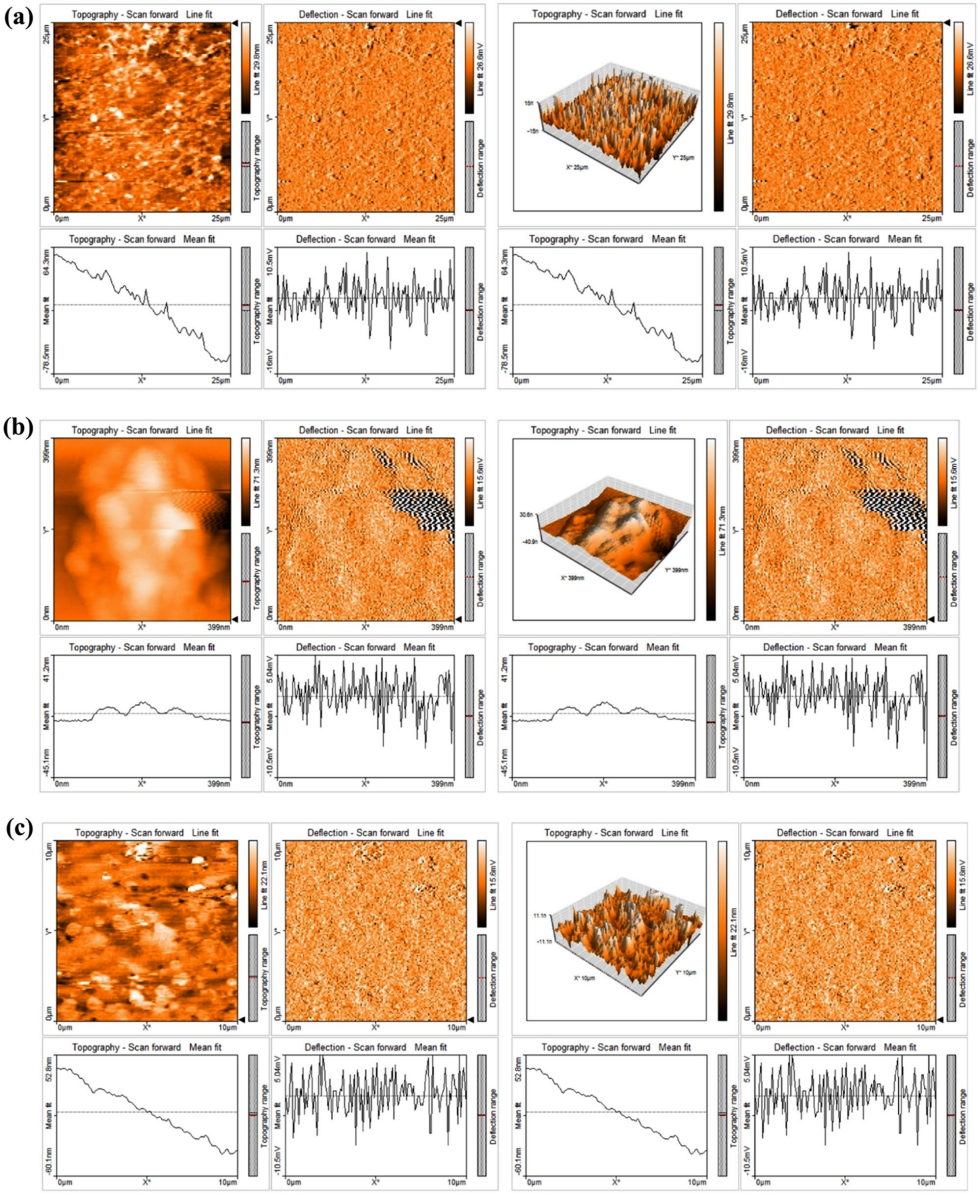
AFM images of INPs synthesized using *Nigella sativa* (**a**) 01:01 (**b**) 01:04 (**c**) 02:04

Atomic Force Microscopy (AFM) was employed to characterize the black cumin seed extract-mediated iron oxide nanoparticles in three-dimensional form with sub-nanometer resolution, as illustrated in Fig. 5. The AFM image clearly depicts the spherical shape of the iron oxide nanoparticles. Spectra obtained from AFM were recorded within ranges of 0–25 μm, 0–399 nm, and 0–10 μm. The spherical shape of iron oxide nanoparticles is significant because it provides a uniform and symmetrical structure that can be advantageous for various applications. Spherical nanoparticles have a high surface area-to-volume ratio, which enhances their reactivity and makes them suitable for catalysis, sensing, and drug delivery [35].

In the context of the provided AFM image, the spherical shape of the iron oxide nanoparticles indicates a uniform size distribution and a symmetrical structure, which can be beneficial for their stability and reactivity. The spherical shape can also facilitate the dispersion of nanoparticles in solutions and their interaction with other materials, making them suitable for applications such as magnetic storage, biosensing, and drug delivery [36].

### Antimicrobial activity

**Fig. 6:**
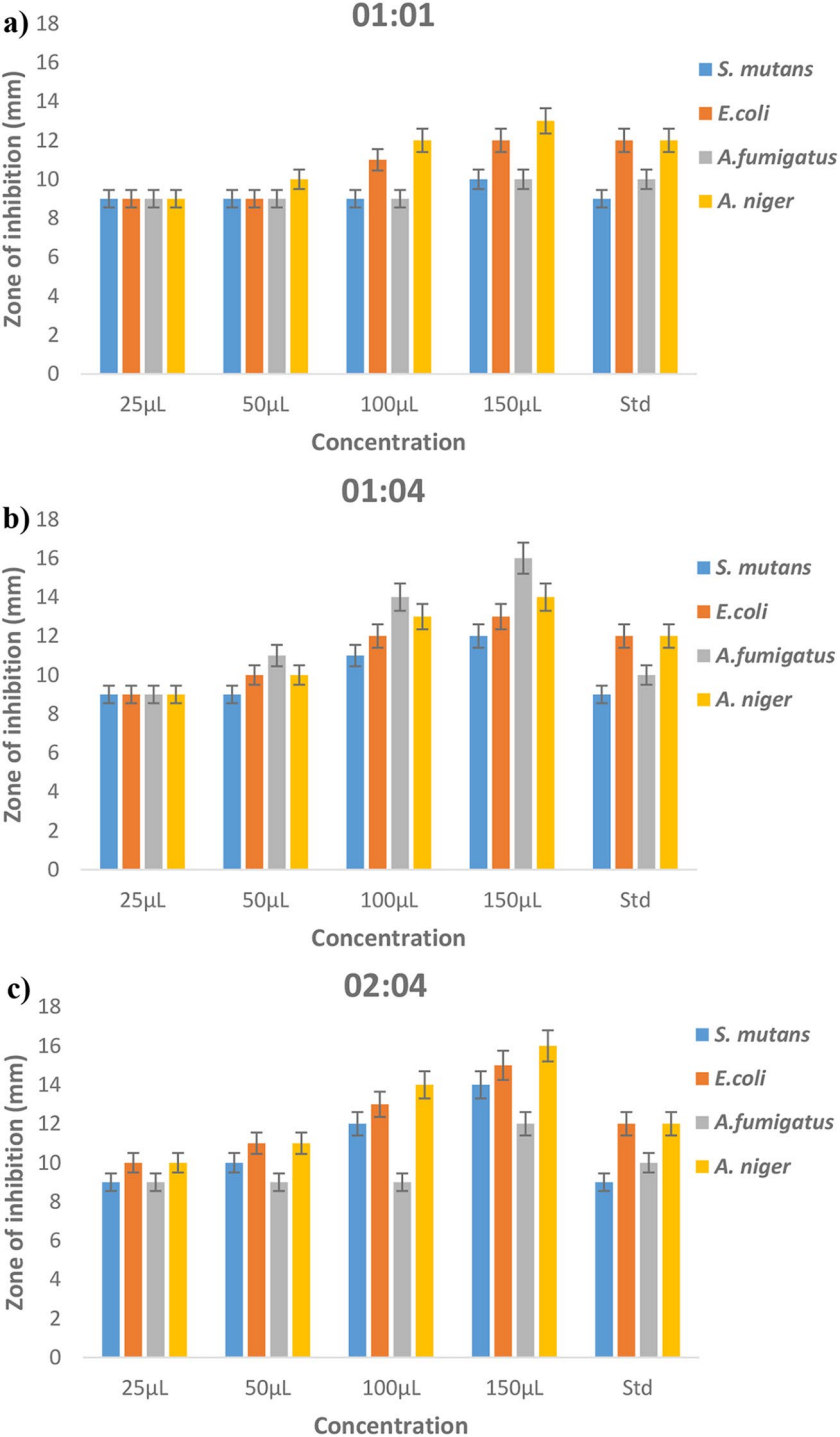
Antimicrobial activity of Black cumin-mediated iron nanoparticles **a**) 01:01 **b**) 01:04 **c**) 02:04

The antimicrobial activity was evaluated using the agar well diffusion technique to assess the efficacy of biosynthesized iron nanoparticles against bacterial pathogens, including Streptococcus spp. and Escherichia coli, as well as fungal pathogens such as Aspergillus fumigatus and Aspergillus niger. Three different ratios of synthesized iron nanoparticles (01:01, 01:04, 02:04) were tested, with each plate containing four concentrations (25 µL, 50 µL, 100 µL, and 150 µL). Tetracycline served as the standard antibiotic for bacteria, while fluconazole was used for fungi. The results, depicted in Fig. 6, demonstrate efficient antimicrobial activity against all pathogens.

The zone of inhibition values varied with changes in nanoparticle ratio and concentration. Generally, higher nanoparticle ratios (01:04 and 02:04) resulted in larger zones of inhibition across all concentrations, indicating enhanced antimicrobial activity. For instance, at 150µL concentration, the 01:04 ratio showed zone sizes of 12 mm (S. mutans), 13 mm (E. coli), 16 mm (A. fumigatus), and 14 mm (A. niger), while the 02:04 ratio exhibited 14 mm (S. mutans), 15 mm (E. coli), 10 mm (A. fumigatus), and 16 mm (A. niger) zones of inhibition. These findings suggest that adjusting the ratio of iron oxide nanoparticles can significantly impact their antimicrobial effectiveness, highlighting their potential as agents for combating microbial growth.

The mechanism behind the inhibition zones of biosynthesized iron nanoparticles involves physical interaction between the cell wall and the nanoparticles, leading to bacterial denaturation and death [37, 38]. The nanoparticles have a positive charge, while microorganisms have a negative charge, leading to electrostatic attraction and interaction with protein thiol groups, releasing ions that disrupt the cell membrane and cause bacterial death. The size of the nanoparticles also affects their toxicity, with larger particles having a different motion from primary nanoparticles, leading to aggregation and clustering, which affects their activity [39, 40].

### Antioxidant activity

**Fig. 7:**
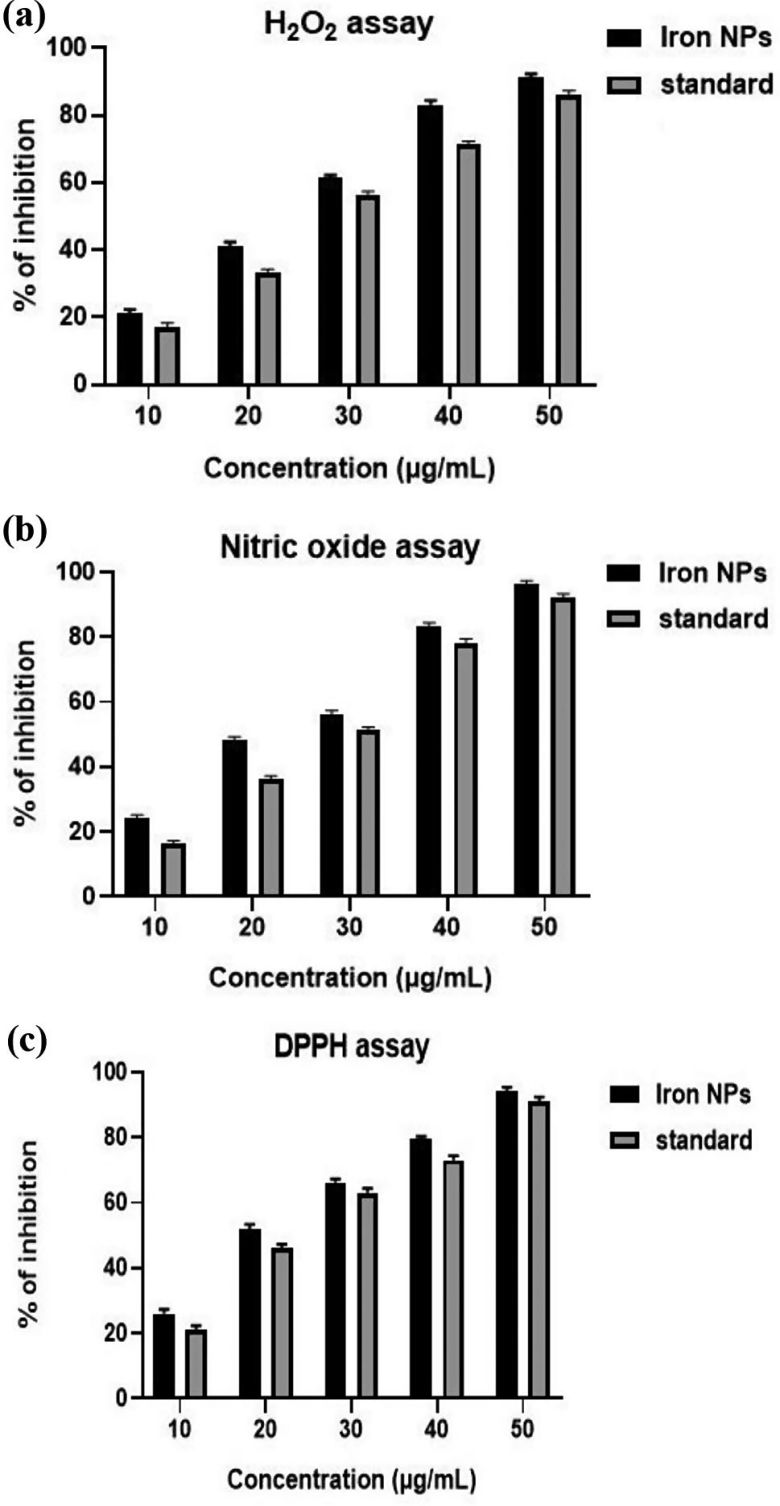
Antioxidant activity of iron oxide nanoparticles using (**a**) H2O2 assay, (**b**) NO2 Assay, (**c**) DPPH assay

In this study, the antioxidant activity of black cumin mediated iron oxide nanoparticles (NPs) was evaluated using three different assays: hydroxyl radical scavenging, nitric oxide radical scavenging, and DPPH radical scavenging. The results showed that the iron NPs exhibited significant antioxidant potential in all three assays when compared to a standard antioxidant (Fig. 7).

In the hydroxyl radical scavenging assay, both iron NPs and the standard antioxidant demonstrated dose-dependent scavenging activity. The iron NPs showed scavenging percentages ranging from 20.65 to 90.54%, while the standard antioxidant exhibited scavenging percentages ranging from 16.32 to 85.45%. This indicates that the iron NPs had a higher scavenging capability for hydroxyl radicals compared to the standard antioxidant at all concentrations tested.

Similarly, in the nitric oxide radical scavenging assay, the iron NPs showed concentration-dependent inhibition of nitric oxide radical production, with inhibition percentages ranging from 23.93 to 95.45%. The standard antioxidant, on the other hand, exhibited inhibition percentages ranging from 15.48 to 91.65%. Once again, the iron NPs displayed higher inhibition of nitric oxide radicals compared to the standard antioxidant across all concentrations.

In the DPPH radical scavenging assay, the iron NPs also demonstrated concentration-dependent inhibition of DPPH radicals, with inhibition percentages ranging from 25.03 to 93.15%. The standard antioxidant showed inhibition percentages ranging from 20.35 to 90.25%. This further confirms the superior antioxidant activity of the iron NPs compared to the standard antioxidant at all concentrations tested in this assay.

Iron oxide nanoparticles with antioxidant properties have potential applications in various fields, including drug delivery, targeted therapy, cancer treatment, and combating oxidative stress. The antioxidant properties of these nanoparticles make them a suitable candidate for targeted delivery, which can open new opportunities for combating oxidative stress in in vivo conditions [41].

In medicine, iron oxide nanoparticles have significant applications, such as drug delivery, targeted therapy, and cancer treatment [42]. They can be used as contrast agents in magnetic resonance imaging (MRI) to improve the visualization of tissues and organs [43]. Additionally, iron oxide nanoparticles can be functionalized with antioxidants, which can enhance their stability and bioactive properties, making them more effective in reducing intracellular reactive oxygen species (ROS) levels in an oxidative stress model induced by hydrogen peroxide (H2O2) [44].

Furthermore, iron oxide nanoparticles can be fabricated using natural antioxidants from plant extracts, such as *Phoenix dactylifera L.* and *Coriandrum sativum L.* leaf extracts, which can exhibit antioxidant activities by TAC and DPPH assays [41]. These nanoparticles can scavenge ROS, making them a promising candidate for combating oxidative stress-related diseases.

Overall, the results of the three assays consistently showed that the black cumin mediated iron oxide nanoparticles exhibited stronger antioxidant activity compared to the standard antioxidant at varying concentrations. This suggests that iron NPs could be a promising candidate for further research and development in the field of antioxidants and could potentially be utilized in various applications for their antioxidant properties.

#### Anti-inflammatory activity

**Fig. 8:**
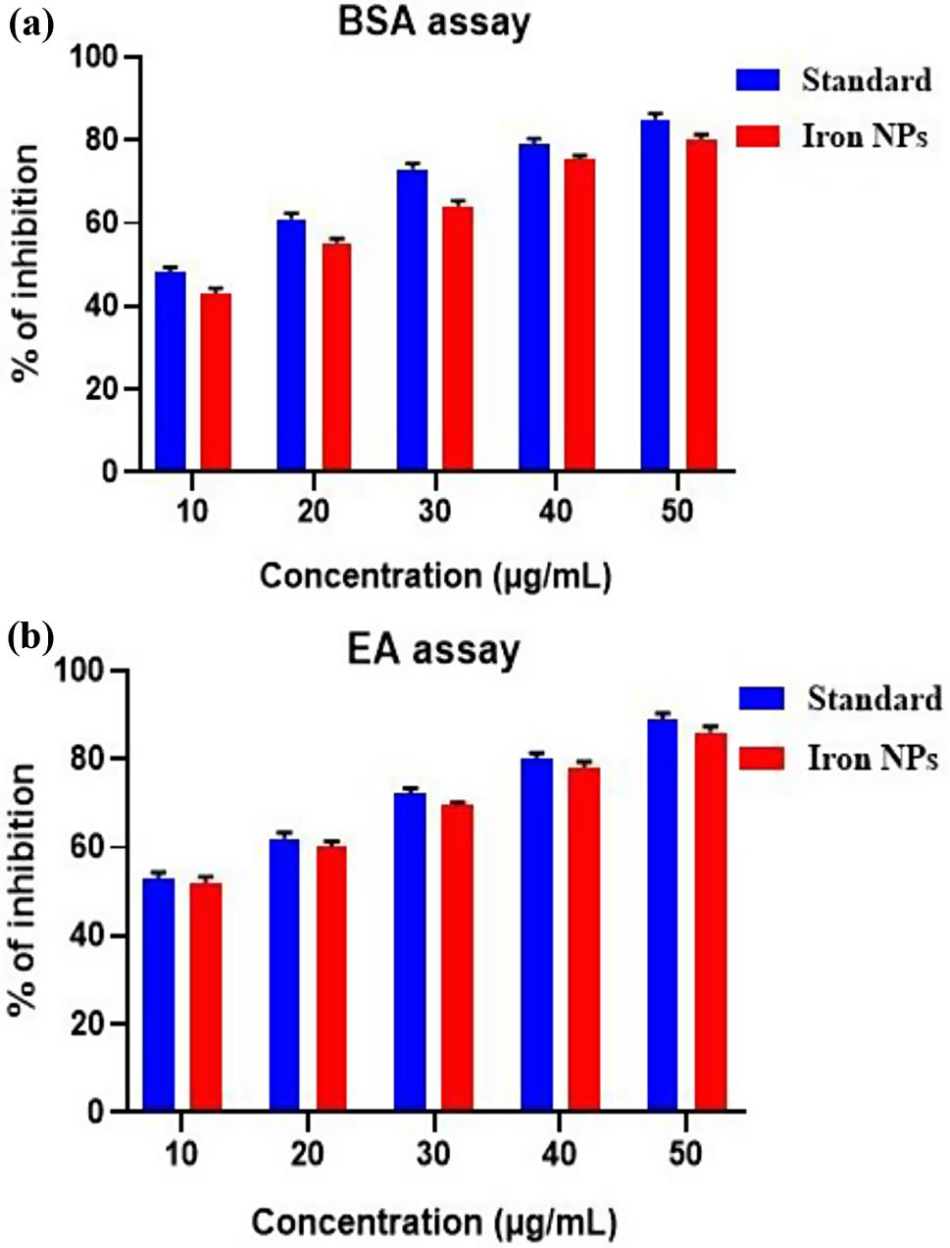
Anti-inflammatory activity using (**a**) bovine albumin denaturation assay and (**b**) Egg albumin denaturation assay

The anti-inflammatory activity of iron oxide nanoparticles was assessed using two different assays, the BSA assay and the EA assay, to evaluate the inhibition of protein denaturation. formation, respectively. In the BSA assay, the percentage inhibition of protein denaturation by iron NPs increased in a concentration-dependent manner, with values ranging from 42.32 to 79.32% at concentrations of 10 µL to 50 µL. The standard anti-inflammatory agent exhibited slightly higher percentage inhibition values, ranging from 47 to 84% at the same concentrations. Similarly, in the EA assay, the percentage inhibition of edema formation by iron NPs also increased with concentration, ranging from 51 to 85%. The standard anti-inflammatory agent showed comparable or slightly higher percentage inhibition values, ranging from 52 to 88% at the same concentrations (Fig. 8).

Previous study reported the synthesis of Fe2O3-NPs using honey as a reducing and capping agent and evaluated their antibacterial, antioxidant, and anti-inflammatory properties. The results showed that Fe2O3-NPs synthesized using honey exhibited significant antioxidant (IC 50 = 22 µg/mL) and anti-inflammatory (IC 50 = 70 µg/mL) activities [45].

Another study found that IONPs can induce oxidative stress in cells by activating pro-inflammatory mediators, which can eventually lead to cell necrosis or apoptosis. This is due to the generation of reactive oxygen species (ROS) by IONPs, which can cause oxidative stress in cells and contribute to their anti-inflammatory effects [46].

Additionally, a study investigating the inflammatory response to nanoparticles, including iron oxide nanoparticles, found that they can involve thrombus formation via the crosstalk to platelets and the coagulation cascade [47]. This suggests that IONPs may have a role in modulating the immune response and inflammation.

Overall, the results of both assays indicate that iron oxide nanoparticles possess significant anti-inflammatory activity, as evidenced by their ability to inhibit protein denaturation. While the standard anti-inflammatory agent showed slightly higher percentage inhibition values in both assays, the iron NPs demonstrated a concentration-dependent increase in activity, suggesting their potential as effective anti-inflammatory agents.

## Conclusion

This study features the new improvements in iron nanoparticle synthesis by black cumin extract. While physical and synthetic techniques for synthesis are more typical, a few eco-accommodating and low-cost synthesis protocols have additionally been created, at times even by utilizing unused plant parts like peels and leaves. Black cumin-mediated iron nanoparticles have been effectively executed in medication and ecological remediation. However, further research has to be carried out in this study to reap its benefits for using it as a biomedical application in future.

## Data Availability

The datasets used and analyzed during the current study are available from the corresponding author upon reasonable request.
